# Malaria Mosquitoes Host-Locate and Feed upon Caterpillars

**DOI:** 10.1371/journal.pone.0108894

**Published:** 2014-11-05

**Authors:** Justin George, Simon Blanford, Matthew B. Thomas, Thomas C. Baker

**Affiliations:** 1 Department of Entomology, Pennsylvania State University, University Park, Pennsylvania, United States of America; 2 Center for Infectious Disease Dynamics, Pennsylvania State University, University Park, Pennsylvania, United States of America; Centro de Pesquisas René Rachou, Brazil

## Abstract

Adult female mosquitoes need blood to develop their eggs and both sexes use nectar and honeydew as carbohydrate resources for flight, survival and to enhance reproduction. However, there are also a few reports in the literature of mosquitoes feeding on haemolymph of soft-bodied insects such as caterpillars. The frequency and significance of this entomophagous behavior is not well understood, but is thought to be a vestige of ancestral feeding behavior or an opportunistic behavior that has evolved over time. In our current paper we investigated the extent to which the malaria mosquito, *Anopheles stephensi*, is attracted to, and can successfully feed on, larvae of two common moth species, *Manduca sexta* and *Heliothis subflexa*. Using y-tube olfactometer assays we found that female *An. stephensi* readily flew upwind to and landed on the caterpillars of both moth species. The nature of the volatile cues used in host location remains unclear but respirometer studies suggest a possible role of CO_2_. Laboratory cage assays further showed that the female mosquitoes were able to actively feed on moth larvae and gain sufficient nutritional benefit to influence survival. The extent to which such an opportunistic behavior occurs in the field has yet to be explored but our results suggest that this haemolymph feeding behavior could play a role in malaria mosquito life history and could provide a novel mechanism for horizontal transmission of pathogens and other micro-organisms between hosts.

## Introduction

Speciation in mosquitoes began to accelerate during the Mesozoic era (65 million years ago), corresponding to the establishment of terrestrial dwellings and nests by birds, mammals and reptiles [1]. Feeding on carbohydrates from flower nectar or honeydew is known to give mosquitoes enough energy for flight, but in order to reproduce they need a blood meal. Adult female mosquitoes are primarily blood-feeders that require blood proteins for developing their eggs [2]. They feed on a wide range of hosts including a variety of vertebrates. Haematophagy in lower dipteran flies is thought to have evolved through a switch in adult feeding behavior from a predatory habit to a bloodsucking one [1], [3], [4]. Mattingly [5] suggested that the ancestors of Culicidae (mosquitoes), which possessed a soft, flexible proboscis not typical of predatory flies, may have originally pierced the skins of soft fruits for nourishment and evolved a blood-feeding habit later. Also, adult males of *Calyptra thalictri* (Lepidoptera: Noctuidae), in a genus that otherwise consists only of adult fruit feeders, exhibit facultative blood feeding behavior that might have evolved from its normal fruit feeding behavior [6]. There is intriguing evidence that haematophagous insects can, and still do, feed on larval stages of other insects, which may have been a part of the evolution toward vertebrate blood-feeding. Triatomine bugs (Hemiptera: Reduviidae) can also feed opportunistically on haemolymph of cockroaches (Blattodea) [7], [8], [9]. The proboscis extension and feeding behavior of triatomine on cockroaches suggests that arthropod haemolymph represents an alternative source of food for triatomines, and they have partially conserved their entomophagous behavior during their evolution to haematophagy [9]. Historic studies report black flies feeding on butterfly pupae [10] and mosquitoes attacking small dipterans and cicadas [11] and caterpillars [12], [13]. This potential for mosquito entomophagy has been generally ignored until recently [14]. The caterpillar feeding behavior of mosquitos' suggest that they have not completely abandoned the entomophagous behavior during evolution and it is still conserved as an opportunistic behavior among dipteran blood feeding insects such as mosquitoes.

In this paper, we investigated whether females of the malaria mosquito *Anopheles stephensi*, were attracted to caterpillars, whether they would feed on them and if so, whether this affected mosquito longevity. We used a Y-tube olfactometer [15], [16] to compare the degree to which *An. stephensi* females were attracted to living vs. dead fourth instar larvae of *Manduca sexta* and *Heliothis subflexa* by landing on or near the caterpillars and feed on them. We then measured the amount of carbon dioxide produced by individual living versus dead caterpillars in order to determine whether there was a significant correlation between caterpillars' CO_2_ output and the amount of upwind flight behavior we had observed in the Y-tube assays. Finally, we placed *An. stephensi* females in cages either containing or lacking live fourth instar caterpillars of *Heliothis subflexa* to measure the degree to which feeding upon caterpillar haemolymph affected mosquito survival. Here we report this opportunistic behavior of *Anopheles stephensi* mosquitoes to fly upwind and land on caterpillars, and the possible visual and olfactory cues they might be using to hostfind and feed on the caterpillars.

## Results

### Olfactometer Choice Assays

**Figure 1:**
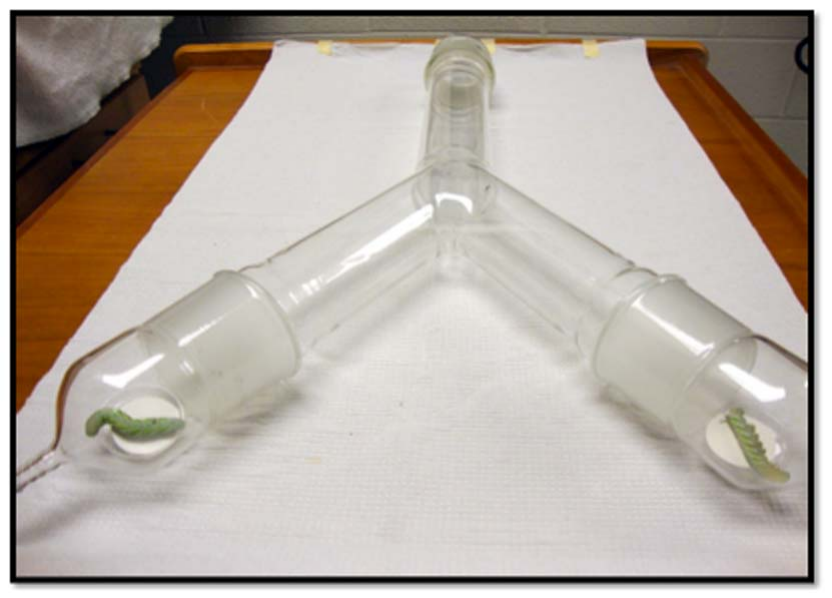
Y-tube olfactometer using live and dead caterpillars. The olfactometer is 70×35×6 cm with the two upwind arms being supplied with charcoal-purified air at a rate of 0.75 litre/sec.

**Figure 2:**
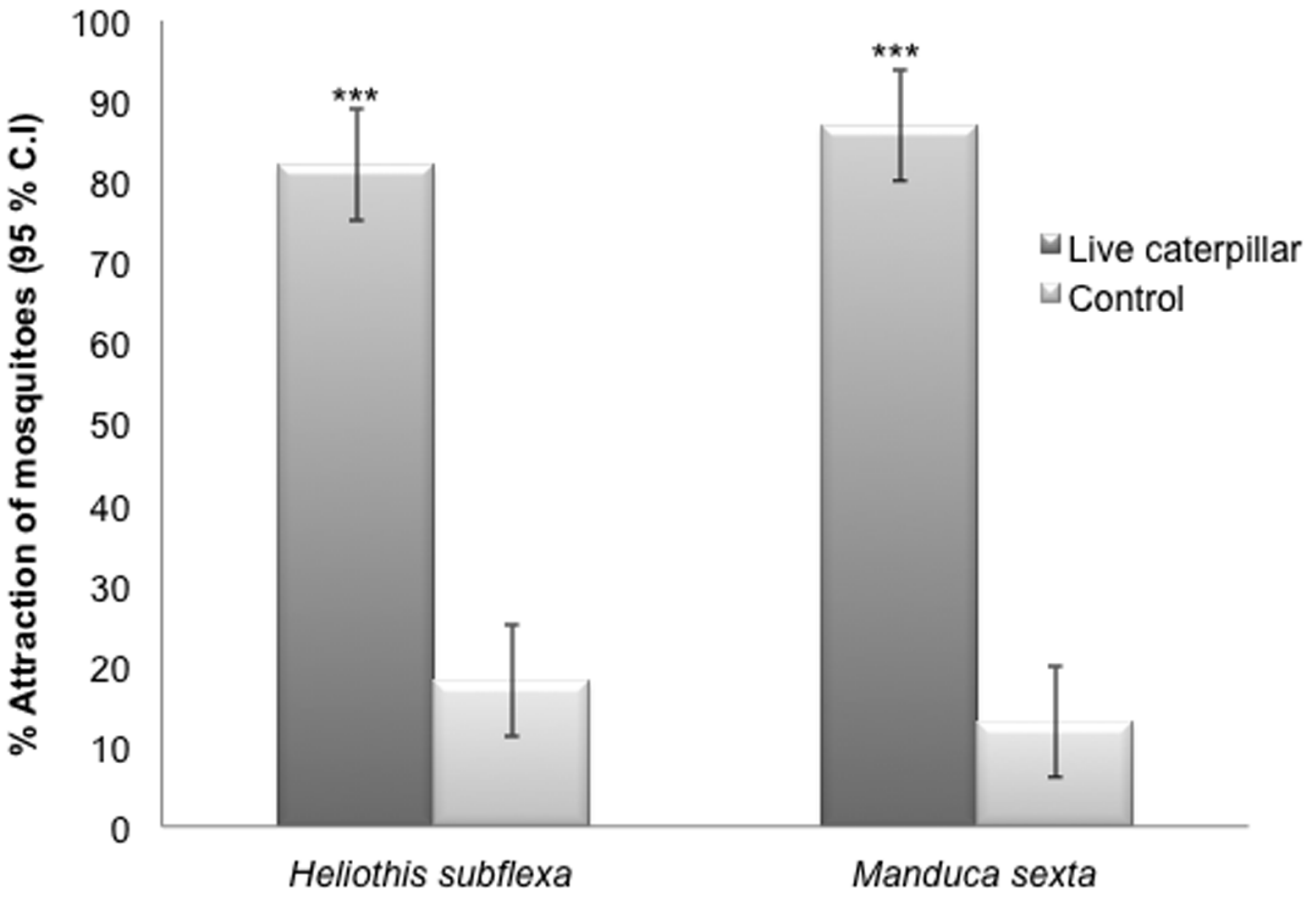
Y-tube olfactometer choice assays showing the percentage of female *An. stephensi* (±95% binomial confidence intervals (C.I.)) that were attracted to live fourth instar larvae of *Heliothis subflexa* and *Manduca sexta* (black bars) versus a white cotton dental wick of the same size used as a blank control (light bars). Asterisks denote significantly different levels of attraction (Chi-square 2×2 test of independence; *P<0.05; **P<0.01; ***P<0.001; N = 90).

**Figure 3:**
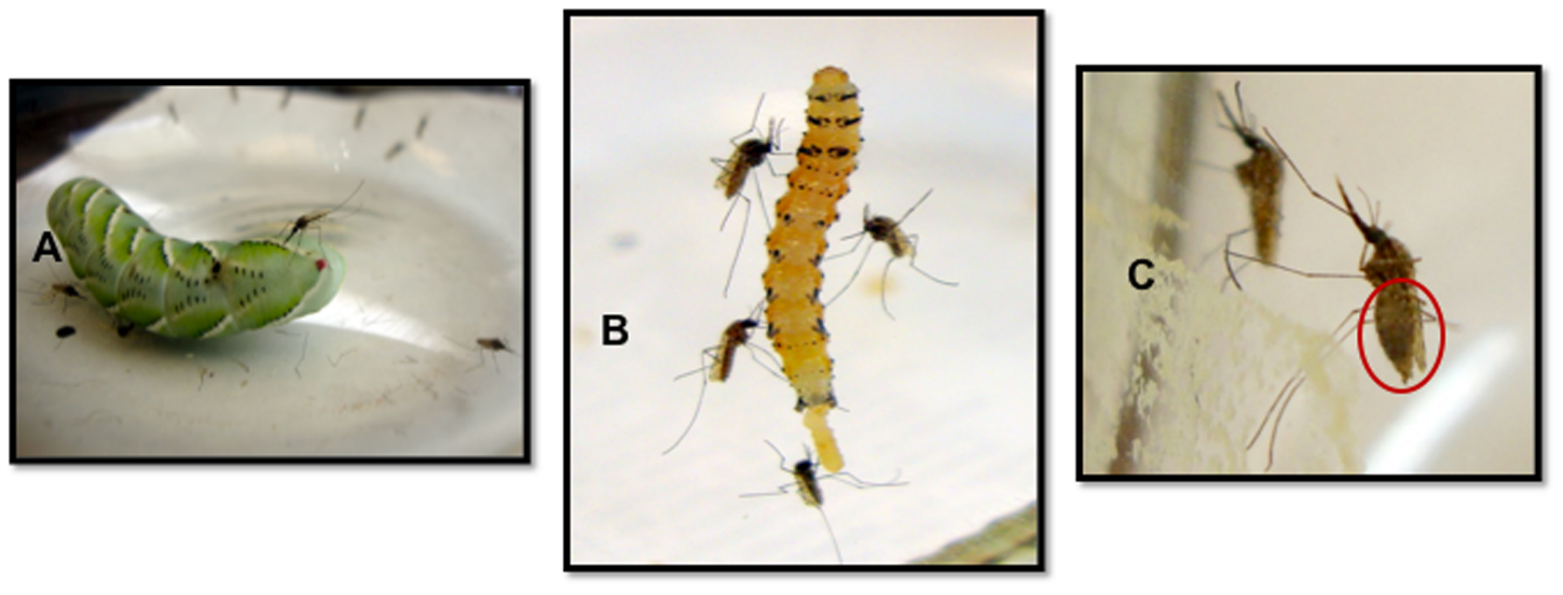
Images show females that had been attracted to, and landed upon for feeding: A. A fourth instar *Manduca sexta*, larva; B. A fourth instar *Heliothis subflexa* larva. **C**. An *An. stephensi* female is shown (foreground) that has engorged on the haemolymph of a *Manduca sexta* caterpillar.

*An. stephensi* females exhibited significant upwind flight attraction responses in the olfactometer assays (**Fig. 1**), flying quickly upwind and landing on or near the caterpillars. Mosquito took only less than 10 seconds to enter the y-tube arm containing the caterpillar and only 15–20 seconds to land on the caterpillar (**Movie S1**-video file). A significantly greater number of mosquitoes was attracted to live *M. sexta* (87%) compared to the clean air cotton wick control arm (13%) (p<0.0001, χ^2^ = 48.4, d.f = 1, N = 90) (**Fig. 2**). In response to *H. subflexa* caterpillars, 82% of the females flew upwind to the live caterpillars compared to 18% flying up the control arm (p<0.0001, χ^2^ = 37.3, d.f = 1, N = 90) (**Fig. 2**). In most cases, mosquitoes started upwind flight within a few seconds and entered the olfactometer arm in less than 10 seconds. Many females landed on the caterpillars and probed them with their mouthparts (**Fig. 3A–B**), with some females becoming engorged on haemolymph (**Fig. 3C**). The caterpillars of both species became agitated and often exhibited defensive behavior to shake off the mosquitoes or else bite them with their mouthparts.

**Figure 4:**
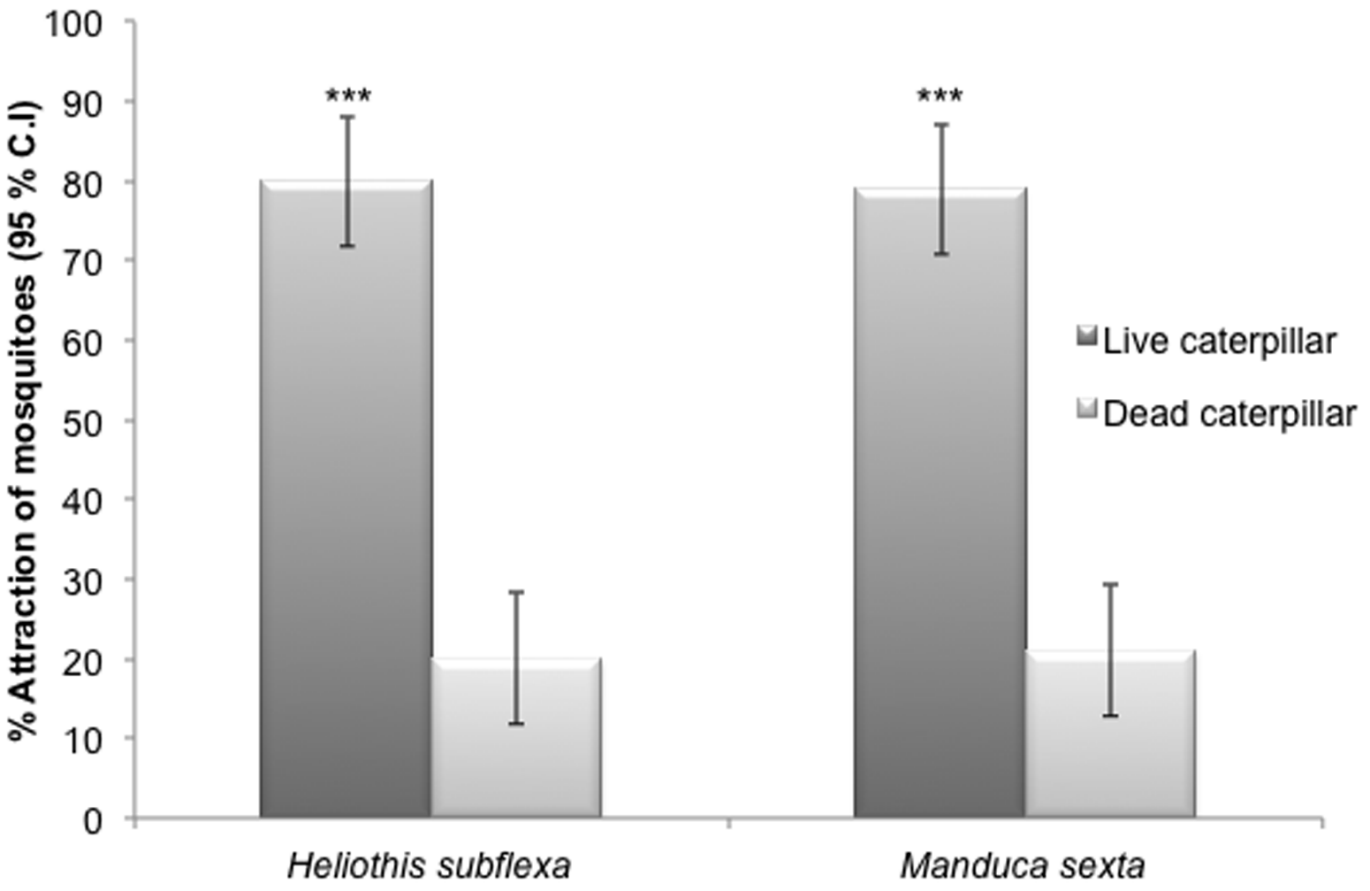
Y-tube olfactometer assays of *An. stephensi* females that were given a choice between live vs. dead caterpillars of two moth species. The figure shows the percentage of female *An. stephensi* (±95% binomial confidence intervals (C.I.) that were attracted to live fourth instar larvae of *Heliothis subflexa* or *Manduca sexta* (black bars) versus dead caterpillars killed by freezing and then thawed back to room temperature (light bars). Asterisks denote significantly different levels of attraction (Chi-square 2×2 test of independence; *P<0.05; **P<0.01; ***P<0.001; N = 90).

In the comparison between live versus dead caterpillars, a significantly greater percentage of *An. stephensi* females flew upwind towards and landed on live caterpillars. In the trials using *H. subflexa*, 81% of the females flew to the arm containing live caterpillars compared to only 19% flying to the dead ones (p<0.0001, χ^2^ = 34.84, d.f = 1, N = 90) (**Fig. 4**). In response to *M. sexta* caterpillars, 79% of females flew upwind to the live caterpillars compared to only 21% to the dead (p<0.0001, χ^2^ = 30.04, d.f = 1, N = 90) (**Fig. 4**). Many of the females landed on the live caterpillars and probed their cuticle (**Fig. 3A–B**). This assay was for measuring attraction to and landing on caterpillars, and so there was no time allowed for females to engorge. However, when *An. stephensi* were given time to feed on these larvae in a closed glass vessel, they exhibited persistent probing and subsequently many became engorged on the caterpillar haemolymph (**Fig. 3C**), as had been previously observed for *Culex* and *Aedes* species [9]–[11].

### Carbon dioxide Measurement using Respirometer Assays

**Figure 5:**
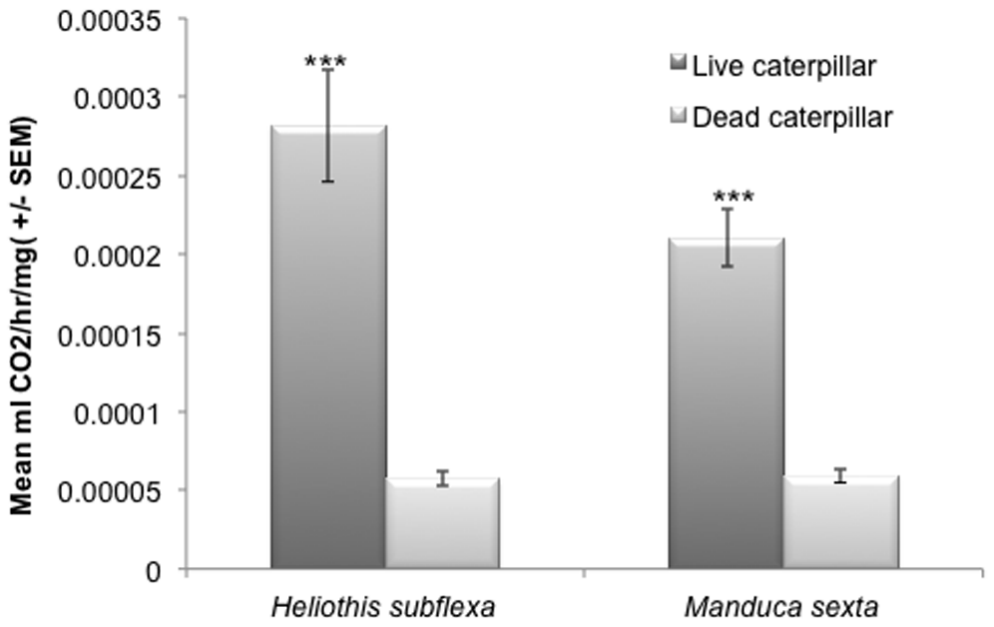
Mean levels of CO_2_ production related to Basal Metabolic Rate (BMR) by live vs. dead caterpillars of two moth species. The mean (± S.E.) amount of CO_2_ (ml CO_2_/hr/gram of body weight) produced by live and dead fourth instar caterpillars of *Heliothis subflexa* and *Manduca sexta* was analyzed using respirometer assays. GLM was used to examine the differences between means for the two treatments (Live vs. Dead, N = 7). The amount of CO_2_ produced by live *M. sexta* caterpillars was significantly higher than that of dead caterpillars (*P*<0.0001; *F*_1, 12_ = 63.59, N = 7). Live caterpillars of *Heliothis subflexa* also produced significantly higher amounts of CO_2_ compared to dead ones (*P*<0.0001; *F*_1, 12_ = 39.34, N = 7).

The respirometer assays measured the amount of CO_2_ produced by individual live vs. dead caterpillars in order to assess this volatile's possible contribution to *An. stephensi* attraction. The measurements showed that the live caterpillars emitted ca. a 10-times greater amount of CO_2_ than did dead caterpillars, as well as a greater mean ml. of CO_2_ per hour per milligram body weight (**Fig. 5**). These live vs. dead values for CO_2_ production were significantly greater for both *M. sexta* (*P*<0.0001; *F*_1, 12_ = 63.59, N = 7) and *H. subflexa* (*P*<0.0001; *F*_1, 12_ = 39.34, N = 7) (**Fig. 5**). Thus CO_2_ emissions from the caterpillars could possibly have contributed to the elevated levels of attraction of *An. stephensi* females to the live compared to dead caterpillars in the upwind flight bioassays.

### Cage Assay to Study the Mosquito Survivability by Feeding on Caterpillars

**Figure 6:**
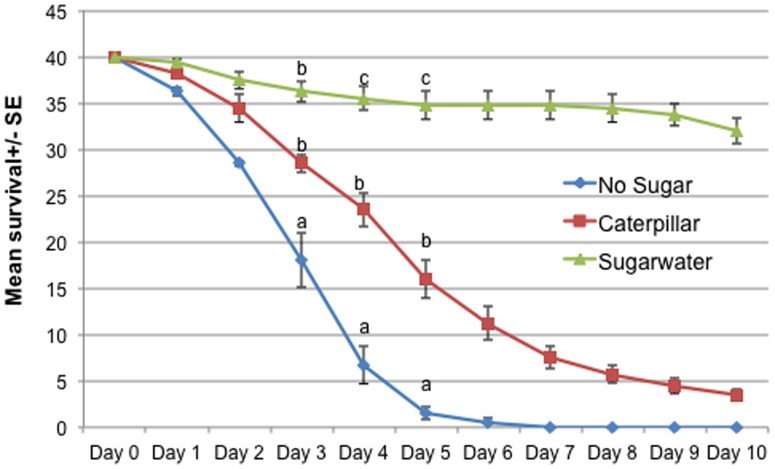
Cage assays examining relative survivorship of female *An. stephensi* feeding on caterpillar haemolymph. Forty mosquitoes were introduced into each cage. The caterpillar treatment cage contained three fourth instar *Heliothis subflexa* caterpillars (N = 160). Treatments were significantly different on day 4 and day 5. GLM was used to examine the difference between treatments on each day. Treatments with different letters above them are significantly different on each separate day.

During the course of this experiment, female mosquitoes were frequently observed landing on the caterpillars and trying to feed on them. Mosquitoes that had access to sucrose water or caterpillars survived longer than those that had no access to food (**Fig. 6**). Treatments were significantly different (χ^2^ = 8.05, d.f = 2, P<0.05). There were no effects for Day (χ^2^ = 8.75, d.f = 4, P = 0.0678) and no Day * Treatment effects (χ^2^ = 9.93, d.f = 8, P = 0.2697) (**Table 1**). Only 40% of the females caged in the presence of caterpillars were dead by Day 4 compared to 85% of those caged with no food. By day 5, mortality of females in the no-food cages had increased to 98%, compared with 60% in cages containing caterpillars. Females provided with continuous access to sucrose water survived the longest, exhibiting only 20% mortality after 10 days.

**Table 1:** Summary of data analysis performed on survival of mosquitoes using different treatments.

Source	df	Chi-square	P-value
Day	4	8.75	0.0678
Treatment	2	8.05	0.0178
Day*Treatment	8	9.93	0.2697
Treatment (Caterpillar vs. Sugar)	1	26.53	<0.0001
Treatment (Caterpillar vs. No Sugar/No caterpillar)	1	50.32	<0.0001
Treatment (Sugar vs. No sugar/No caterpillar)	1	85.76	<0.0001

Assays were done for 10 days. Data fit to a binomial model describing the probability of a mosquito surviving each day. Proc Genmod was used in SAS where Day and Treatment (feeding status) were the main effects. The repeated statement was used to adjust for correlations within replicates. Ward Chi Square statistics were generated. Pairwise comparisons were conservatively adjusted using a Bonferoni correction. Data past day 5 were not included because unfed caterpillars no longer exist. Treatments were significantly different (P<0.05).

## Discussion

Our study shows that females of the malaria mosquito *An. stephensi* are attracted to, and opportunistically feed upon, two different species of caterpillars, and survive better when they have access to caterpillars as possible food sources than when they are deprived of food completely. Two prior studies have shown that females of two other mosquito species will readily feed upon soft-bodied insects including caterpillars [12]–[13] and gain enough nutrition to lay viable eggs [13]. A third study recently showed that mosquito-feeding on caterpillars is sufficiently detrimental to the caterpillars that it reduces their fitness [14]. A recent study by our group has also shown that *An. stephensi* females were attracted to caterpillars infected by fungal pathogens [16] but did not directly examine the extent of feeding. This study showed that mosquitoes can fly upwind to the olfactory cues emanating from fungal spores and they pick up spores and get infected with the *Beauveria bassiana* fungus. The infected mosquitoes died in 4–6 days and sporulated and these sporulating cadavers elicited upwind flight behavior from other mosquitoes in Y-tube olfactometer choice assays [16].

Very few studies [12], [13], [14] have looked at this unusual behavior of mosquitoes which might have been partially conserved as an opportunistic behavior during the evolution from entomophagy to haematophagy. None of these previous studies have conducted choice discriminating assays such as olfactometer choice assays to study their upwind flight behavior or have not looked at the specific cues mosquitoes' use for finding the caterpillar host. In this study we show that mosquitoes can detect the CO_2_ and other heat cues emanating from live caterpillars compared to dead caterpillars and can use them along with visual cues to land on them and feed on them. Our results show that significantly higher numbers of mosquitoes were alive on days 3 to 6 and the following days compared to no sugar water/starved condition. Cage assays shows that mosquitoes are able to survive longer by feeding on caterpillars under no food or nectar conditions and this opportunistic feeding behavior of mosquitoes can provide them some nutrition when nectar feeding resources are not readily available. However, observations from the relevant cages revealed that many mosquitoes were actually being killed by the caterpillars. Thus, it is difficult to determine whether the sugar-fed mosquitoes survived better because of nutritional factors alone, or whether reduced risk of mortality due to caterpillar defensive behaviors (and even predation), also played a role.

Healthy caterpillars evoked substantial levels of upwind flight attraction of *An. stephensi* mosquitoes that was correlated with the higher CO_2_ emission rates from living compared to dead caterpillars. In nature, female mosquitoes face the obligatory task of finding suitable protein resources for egg production, and they use their olfactory system to respond to different vertebrate host-odor cues. CO_2_ plays a significant role in activating and orienting the females of most species to fly upwind over long distances to locate their hosts [17], [18], using volatile co-attractant factors as well, such as lactic acid [19] and 1-octen-3-ol [20], [21]. Our analysis of dead vs. live caterpillar CO_2_ emission indicated that the greater amount of attraction to the living caterpillars was correlated with a 10-fold higher emission of CO_2_. The amount of CO_2_ produced by caterpillars in nature could very well be at behaviorally detectable levels for mosquitoes, and could act as a significant volatile co-factor involved in the location of caterpillars, just as it is in the orientation of mosquitoes toward vertebrate hosts.

The degree of caterpillar feeding by mosquitoes in nature has not yet been established, but it is an important opportunistic behavior by female mosquitoes that warrants further research considering their ability to vector pathogens of human diseases. Mosquitoes derive sugar from diverse plant sources [22], [23] and many species spend time resting outdoors [24] so it is possible that they are in close proximity to plant-feeding caterpillars. Thus, the potential for opportunistic feeding on caterpillars might be high. Such behavior could provide alternative food resources that might be especially important when availability of nectar or fruit sugars is limited. Additionally, by feeding on insect larvae, mosquitoes might act as vectors of insect diseases and perhaps transfer viruses and other microorganisms (such as *Wolbachia*) from insect to insect, or even from insect to vertebrate hosts. These events could be ecologically and evolutionary important, even if rare. More research needs to be done using natural populations to examine how much, if any, feeding is performed upon soft-bodied insect larvae by mosquitoes in the field. If such feeding was indeed a basal trait before the shift to blood-feeding on vertebrates occurred, it would be important to know whether or not it has been entirely abandoned by present-day mosquitoes.

## Materials and Methods

### Mosquito Rearing

*An. stephensi* Liston were reared under standard insectary conditions of 27°C, 80% humidity and 12:12 light: dark photo-period. Eggs were placed in plastic trays (25 cm×25 cm×7 cm) filled with 1.5 l of distilled water. To reduce variation in adult size at emergence, larvae were reared at a fixed density of 400 per tray. Larvae were fed Liquifry for five days and then on Tetrafin fish flakes (Tetra, Virginia, USA). From approximately two weeks after egg hatch, pupae were collected daily and placed in emergence cages. The adults that emerged were fed *ad libitum* on a 10% glucose solution. All experiments used four to six day old adult female mosquitoes. The sugar solution was removed four hours before the mosquitoes were used in the experiments.

### Caterpillar Rearing

Fourth instar larvae of *H. subflexa* or *M. sexta* were used in the experiments, because they are representative of the cosmopolitan moth families Noctuidae and Sphingidae whose caterpillars *An. stephensi* females might easily encounter in their natural habitats in Asia. Laboratory colonies of these species were maintained on artificial diet under controlled conditions (14:10 h L: D, 23°C, 60% relative humidity) in a rearing room. The eggs of tobacco hornworm (*M. sexta* L.), were obtained from Carolina Biological Supply (Burlington, NC, USA) and allowed to hatch in Petri dishes (90 mm×15 mm) on moist filter paper (Whatman 1; 90 mm) in a growth chamber (16 : 8 L : D; 25°C: 22°C Day: Night; 65% RH). Larvae were then moved to plastic containers (35 cm long×10 cm high×15 cm wide) in the same growth chamber for mass rearing on artificial casein diet. Late third instars of *M. sexta* were used in the Y-tube olfactometer assays and for CO_2_ measurements. Late third instars of *H. subflexa* were used in olfactometer assays, CO_2_ measurements and in the cage assays to assess the survivability of *An. stephensi* by feeding on caterpillars. Caterpillars were removed from the diet cups one hour before using in the experiments and all the surface excrements were removed before using them in the experiments.

### Olfactometer Choice Assays

A Y-tube olfactometer (70×35×6 cm) was used for the behavioral assays (**Fig. 1**). Only female mosquitoes were used in the olfactometer assays as male mosquitoes showed poor upwind flight behavior to caterpillars or to other chemical odorants. Female *An. stephensi* were released at the main, downwind end of the Y-tube with charcoal-purified air pushed through both arms of the Y-tube at the rate of 0.75 litre/sec. One 4^th^-instar laboratory-reared larva of either *H. subflexa* or *M. sexta* was placed on a cardboard disc and retained inside the arms of the Y-tube at the extreme upwind end. Mosquitoes were assayed individually and those that flew upwind the length of the tube and landed on or near the caterpillars or in the control arm (cotton dental wick of the same dimension caterpillar) were removed using an aspirator and never used again. The orientation of the arms of the Y-tube were flipped 180° after 15 individual flights to avoid lighting-related bias, and tube's interior walls were rinsed with hexane at that time. Four-to-six day old lab-reared female *An. stephensi* were used in the study. Females that did not initiate an upwind flight after two minutes were discarded. Choice assays were performed using live caterpillars vs. a blank (dental wick) control, and in addition, live caterpillars were compared against dead caterpillars that had been frozen for 12 hours at −20°C, and then thawed back to room temperature.

### Carbon dioxide Measurement using Respirometer Assays

Metabolic rates were measured by placing each live or dead caterpillar into its individual flow-through respirometry chamber as explained in Blanford et al. [25]. Dry CO_2_-free air was passed through the 20 ml chambers at 0.25 litres/min and then dried and passed through a Li-Cor 6252 CO_2_ analyzer. Within each run, seven experimental chambers containing an individual caterpillar were sampled in sequential fashion by using a computer-controlled valve system. Three chambers each contained a live caterpillar and four contained a dead caterpillar; these were used in the first run and the order was reversed for the second run giving 7 replicates. An eighth chamber was left empty and sampled between each of the occupied chambers to establish a baseline. All chambers were housed in a reach-in incubator set to 25 (±0.2)°C. Analog signals from the flow meter and carbon dioxide analyzer were converted to digital and recorded on a computer (Sable systems, Salt Lake City). The amount of CO_2_ produced by live and dead caterpillars were measured. The caterpillars were used only once in the experiments.

### Cage Assay to Study the Mosquito Survivability by Feeding on Caterpillars

Female *An. stephensi* that were four to six days old were used for the study. The mosquitoes were never blood-fed, and were given access to 10% sugar solution until the start of the experiment. Forty mosquitoes were introduced into a rectangular plastic cage (30 cm×15 cm×15 cm) that had a removable top covered with a mesh that allowed free passage of air. The experiment consisted of three treatments and four replicates for each treatment (N = 160). In the first treatment, mosquitoes were provided with 10% glucose solution in a 40 ml glass bottle with filter paper wicking the sugar solution for mosquitoes to access to the wick. In the second treatment, three 4^th^ -instar *H. subflexa* larvae were placed inside a second cage and allowed to roam around the cage. A previous study of mosquito feeding on caterpillars [10] had used caterpillars that had been either coddled or else had been tied to the sides of the cages, which prevented the free movement of caterpillars in the cage. No food or water was provided to the caterpillars as this could have provided nutrition for the mosquitoes as well. *H. subflexa* caterpillars were changed every 24 hours with new freshly fed caterpillars. In the third treatment group mosquitoes received no glucose or any other form of nutrition for the duration of the experiment. All cages were kept under 14: 10 light-dark cycle and their positions were randomly rotated each day. The experiments were run until all the mosquitoes in the caterpillar cage were dead, or else until 10 days had elapsed and mosquito mortality was monitored daily. Data were analyzed using SAS and Chi-square 2×2 tests of independence. The cage assays for mosquito survival were analyzed using Wald Chi Square statistics. Pairwise comparisons were conservatively adjusted using a Bonferoni correction.

## Supporting Information

Movie S1 **Video showing the upwind flight behavior of female** ***An. stephensi*** **mosquito towards a fourth instar larvae of** ***Manduca sexta*** **Mosquito took only 6 seconds to enter the y-tube arm containing the caterpillar and only 20 seconds to land on the caterpillar.**
(MPG)
